# Secondary Degeneration of White Matter After Focal Sensorimotor Cortical Ischemic Stroke in Rats

**DOI:** 10.3389/fnins.2020.611696

**Published:** 2021-01-18

**Authors:** Zhaoqing Li, Huan Gao, Pingmei Zeng, Yinhang Jia, Xueqian Kong, Kedi Xu, Ruiliang Bai

**Affiliations:** ^1^Interdisciplinary Institute of Neuroscience and Technology, School of Medicine, Zhejiang University, Hangzhou, China; ^2^Key Laboratory of Biomedical Engineering of Education Ministry, College of Biomedical Engineering and Instrument Science, Zhejiang University, Hangzhou, China; ^3^Qiushi Academy for Advanced Studies (QAAS), Zhejiang University, Hangzhou, China; ^4^Department of Chemistry, Zhejiang University, Hangzhou, China; ^5^Department of Physical Medicine and Rehabilitation, The Affiliated Sir Run Run Shaw Hospital, School of Medicine, Zhejiang University, Hangzhou, China; ^6^Zhejiang Provincial Key Laboratory of Cardio-Cerebral Vascular Detection Technology and Medicinal Effectiveness Appraisal, Zhejiang University, Hangzhou, China

**Keywords:** secondary degeneration, white matter, diffusion tensor imaging, microstructure MRI, photothrombotic ischemia, sensorimotor cortex

## Abstract

Ischemic lesions could lead to secondary degeneration in remote regions of the brain. However, the spatial distribution of secondary degeneration along with its role in functional deficits is not well understood. In this study, we explored the spatial and connectivity properties of white matter (WM) secondary degeneration in a focal unilateral sensorimotor cortical ischemia rat model, using advanced microstructure imaging on a 14 T MRI system. Significant axonal degeneration was observed in the ipsilateral external capsule and even remote regions including the contralesional external capsule and corpus callosum. Further fiber tractography analysis revealed that only fibers having direct axonal connections with the primary lesion exhibited a significant degeneration. These results suggest that focal ischemic lesions may induce remote WM degeneration, but limited to fibers tied to the primary lesion. These “direct” fibers mainly represent perilesional, interhemispheric, and subcortical axonal connections. At last, we found that primary lesion volume might be the determining factor of motor function deficits.

## Introduction

Stroke is one of the leading causes of death and disabilities (Krishnamurthi et al., 2015) and produces functional deficits resulting from neuronal death in primary lesion areas and from possible secondary degeneration of surrounding or remote regions. Acute neuroprotection to prevent tissue damage within the peri-infarct region and to reduce the final infarct volume is a focus of clinical research (Majid, 2014). Indeed, both clinical studies on stroke patients and animal studies with stroke models reveal the critical role of infarct volume on functional outcome (Peeling et al., 2001; Roof et al., 2001; Zaidi et al., 2012; Vagal et al., 2015; Turner et al., 2016). In addition, accumulating clinical evidence has shown that delayed secondary degeneration also occurs in remote non-ischemic cortical regions (Duering et al., 2015; Wei et al., 2019). Axonal connectedness is believed to be the key linking remote neuronal damage to the primary lesion site (Dikranian et al., 2008), suggesting that potential white matter (WM) degeneration also occurs after stroke. The use of light and electron microscopic methods in early animal studies has shown that WM can be damaged by focal cortical ischemia (Pantoni et al., 1996). Using non-invasive neuroimaging tools, secondary degeneration of WM after ischemia has been shown in several human studies (Lindberg et al., 2007; Yu et al., 2009; Egorova et al., 2020). Although the role of secondary degeneration in stroke recovery has not been well understood, emerging clinical studies suggest that secondary WM degeneration is associated with neurological deficits and can predict functional outcome after stroke (Puig et al., 2010; Yin et al., 2013; Guo et al., 2017). More importantly, several neuroprotective agents have been proposed and have demonstrated efficacy in reducing secondary degeneration and improving functional outcome in animal models (Jian et al., 2019), suggesting the potentially important roles of secondary degeneration in poststroke recovery.

As awareness of the importance of secondary degeneration after stroke increases, the first question that needs to be answered is where the secondary degeneration occurs and its relationship to the primary ischemic lesion. The mainstream assumption is that secondary degeneration occurs primarily on cortical/subcortical regions that have direct synaptic connections with the primary lesion area, but conclusive evidence is lacking for this assumption. This assumption primarily comes from histopathological findings in preclinical studies (Jones et al., 2015; Weishaupt et al., 2016; Cao et al., 2020), but such studies suffer from limited fields of view and the lack of information concerning axonal connections. Recently, several advanced neuroimaging tools [diffusion MRI (Basser et al., 1994; Alexander et al., 2019), myelin imaging (Laule et al., 2007)] with the capability to characterize whole-brain structural axonal connections and microstructure properties along axonal connections have been developed. With these emerging neuroimaging tools, evidence of secondary WM axonal degeneration, such as Wallerian degeneration in the remote corticospinal tract, has been found in human studies (Lindberg et al., 2007; Yu et al., 2009; Guo et al., 2017). However, human studies suffer from diverse localization and size of the primary ischemic lesion and the challenges in performing statistics with such diverse lesions. In contrast, animal models with well-controlled stroke lesion, together with ultra-high-field neuroimaging tools to characterize whole-brain axonal connections and tissue degeneration, would be an ideal way to systematically study the spatial distribution of secondary WM degeneration. The ultra-high-field magnets with its higher signal-to-noise ratio (SNR) and spatial resolution could greatly enhance our ability to use diffusion tensor imaging (DTI) to study microstructure more exquisitely in a whole-brain level. However, animal studies on secondary WM damage using whole-brain neuroimaging tools are still few (Jung et al., 2017; Aswendt et al., 2020), and their relation to primary ischemic lesions and functional outcomes have not yet been well understood.

In this study, we systematically explored secondary WM degeneration following induced focal sensorimotor cortex ischemia in rats using microstructure MRI, primarily diffusion MRI with high and isotropic resolution. The comparable plane but highly improved resolution in slice direction compared with previous *ex vivo* and *in vivo* studies (Jiang et al., 2006; Aswendt et al., 2020) could help track WM more exquisitely and be beneficial for the quantitative assessments of WM secondary degeneration. The importance of isotropic resolution for fiber tractography has been demonstrated in some studies (Mukherjee et al., 2008; Neher et al., 2013). Focal ischemic injury of the sensorimotor cortex is a well-established small-lesion model that has been widely used in experimental animals to study the underlying neurological mechanisms of motor function deficits and recovery (Stroemer et al., 1995; Dancause et al., 2006; Nudo, 2006). Several issues were addressed in this study: (1) the spatial distribution of the secondary damage on WM, (2) the fiber tracts which undergo degeneration, and (3) the role of the primary lesion and secondary WM damage in determining the final motor function outcome. For these purposes, high-resolution diffusion MRI using a 14 T microstructure MRI system was performed on *ex vivo* rat brains to explore whole-brain WM microstructure changes. Focal unilateral sensorimotor cortical ischemia was induced in rat brains using the photothrombotic ischemia (PTI) method (Labat-gest and Tomasi, 2013). Then, tract-based spatial statistics (TBSS) (Smith et al., 2006) was used to explore the microstructure changes in whole-brain WM after 4–5 weeks of spontaneous recovery following ischemia. By projecting DTI-derived parameters (FA, fraction anisotropy) onto a FA skeleton—which represents the centers of all tracts common across subjects—TBSS alleviates residual image misalignment and allows for whole-brain searching of WM tissue showing axonal degeneration without a preliminary hypothesis about specific tracts or regions of interest (ROIs). In addition, fiber tractography was performed to study the axonal connection properties of these abnormal WM sites. Finally, the relation between the primary lesion volume, the secondary WM degeneration, and final motor functional outcome was investigated.

## Materials and Methods

### Focal Sensorimotor Cortical Ischemic Lesion in a Rat Model

All surgical and experimental protocols in this study were carried out in accordance with the Guide for The Care and Use of Laboratory Animals (China Ministry of Health) and approved by the Animal Care Committee of Zhejiang University, China. Fourteen adult male Sprague–Dawley rats, who were 2–3 months old and weighed between 230 and 280 g, were included in this study. All rats were housed in individual cages under a 12/12 h light/dark cycle with free access to water. Food was restricted to 15 g chow per day to motivate reaching and grasping performance during the training and testing periods. Eight rats were subjected to the surgery and lesion group. Based on a protocol in a previous study (Labat-gest and Tomasi, 2013), we used the PTI model to induce ischemia on the sensorimotor cortex contralateral to the dominant forelimb. Briefly, rats in the lesion group were anesthetized with propofol (20 ml:200 mg), and a 4 mm-wide squared craniotomy was prepared on the cortex contralateral to the dominant forelimb (centered at 3.5 mm lateral and 0.5 mm anterior to the bregma). The skull was carefully removed with the dura kept intact. Rose Bengal solution (1 ml, 15 mg/ml) was intravenously injected into the tail vein 2 min before laser irradiation. Ischemia was induced by focal illumination (25 mW) of a 532 nm laser (CNI Laser, Changchun, China) for 15 min with a 3.5 mm-diameter mask over the craniotomy window. After laser illumination, the skull was covered using medical adhesive. Six rats without surgery served as the control group.

The single-pellet retrieval (SPR) task was adopted for the assessment of precise reaching and grasping behavior (Alaverdashvili and Whishaw, 2013). Before surgery, all rats in the lesion group were trained for this SPR task until their success rates were above 60% for 3 continuous days. The motor functional behavior was further tested at 1 day before ischemia and 2, 3, 4, 6, 8, 10, 12, 14, 16, 18, 20, and 21 days after ischemia. On testing days, rats underwent a session of an SPR task consisting of 40 trials or lasting 15 min. The number of successful reaches divided by the total number of reaches was calculated as the success rate. For each rat, we defined the ratio of success rate after ischemia to the success rate at 1 day before ischemia as the motor behavioral score for that day. At 4–5 weeks after ischemia, all rats were sacrificed with 30% chloral hydrate overdose and transcardially perfused with 0.9% saline solution and 10% formaldehyde solution. The brains were then postfixed in 4% paraformaldehyde.

### *Ex vivo* MRI Acquisition

All scanning was performed on a 14 T vertical Bruker Micro imaging system with a 30 mm RF coil. The *ex vivo* brains were rehydrated in phosphate-buffered saline for at least 3 days before imaging and immersed in perfluoro polyether (FOMBLIN^®^, Solvay Specialty Polymers Italy SpA) to avoid magnetic susceptibility artifacts during MRI scanning. Diffusion weighted image (DWI) was achieved using a three-dimensional echo planar imaging (EPI) sequence with eight segments: TE/TR = 31/800 ms, *b* = 3,800 s/mm^2^, 32 gradient directions, *δ*/*Δ* = 3/18 ms, 0.16 × 0.16 × 0.16 mm^3^ voxel size, and two acquisitions at *b* = 0 s/mm^2^. Here, the *b* values were chosen to induce similar diffusion signal attenuation as conventional DTI *in vivo* (*b* around 1,000 s/mm^2^) as the diffusivity in *ex vivo* brain is around 3–4 times smaller than that in *in vivo* brain. Similar *b* values were also used by other groups for *ex vivo* studies (Irfanoglu et al., 2016; Haber et al., 2017). DWIs with the opposite phase-encoded directions were acquired at *b* = 0 s/mm^2^ for geometric distortion correction for EPI. The total time for DWI acquisitions was 7 h 20 min 58 s. *T*_2_-weighted images were obtained using a three-dimensional multislice multi-echo sequence (MSME) with the same spatial dimensions as DWIs: TE/TR = 4/1,000 ms and 32 echoes.

### MRI Data Preprocessing and Microstructure Metrics Calculation

**FIGURE 1:**
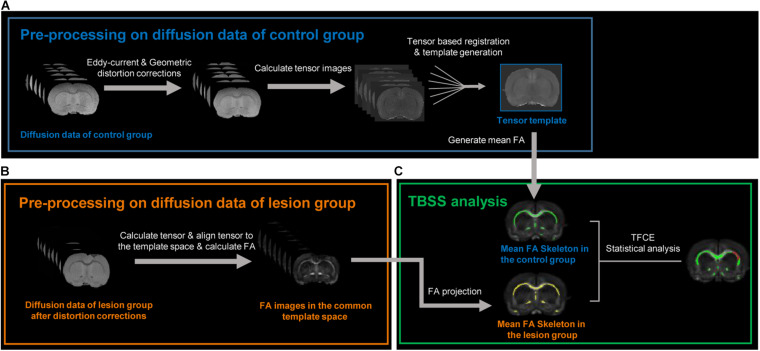
Image processing pipelines used in this study. **(A)** Diffusion data preprocessing of the control group. After eddy current and geometric distortion corrections, the preprocessed diffusion data in the control group were used to calculate tensor images. All tensor images in the control group were aligned with each other to transform into a common space and generate a tensor template and a mean FA (fractional anisotropy) image. **(B)** After the same distortion corrections, the diffusion data of the lesion group were also used to calculate respective tensor images, aligned into the above tensor template via tensor-based registration, and generated respective FA image in the common template space. **(C)** Tract-based spatial statistic (TBSS) analysis was used to compare FA difference between the control and lesion group at whole-brain level in the common space. The green and yellow bundles, respectively, represent the FA skeleton in the control and lesion groups. The red bundles indicate the skeletons with decreased FA in the lesion group compared with the control group after an unpaired *t*-test with threshold-free cluster enhancement (TFCE) and family-wise error correction.

Figure 1 illustrates the pipeline for MRI data preprocessing and TBSS analysis in this study. All DWI preprocessing—including eddy current and EPI geometric distortion corrections, diffusion tensor image registration, and diffusion tensor template generation—was done using TORTOISE software (Pierpaoli et al., 2010). For datasets of the control group, first, AC–PC alignments were performed on the *T*_2_-weighted structural images within MIPAV^1^ to provide a preliminary initialization for the following tensor template generation. The *T*_2_-weighted images after AC–PC alignments were further used as reference images for eddy current and EPI geometric distortion corrections along with the DWIs of the opposite phase encoding direction using the DIFFPREP and DRBUDDI (Irfanoglu et al., 2015) tools in TORTOISE. Then, the preprocessed DWIs of each subject in the control group were fitted to the non-linear DTI model (Basser et al., 1994) to generate diffusion tensor images. Next, all diffusion tensor images in the control group were used to generate a tensor template with the DRTAMAS (Irfanoglu et al., 2016) tool in TORTOISE, which is a framework for intersubject registration and template creation from DTI datasets. The registration methods used in DRTAMAS resulting in the simultaneous deformation of a population of subject images (here, it is all tensor images in the control group) into a new average image that evolves iteratively. The final averaged tensor image will serve as the template to represent the population group in this study. Finally, the FA image of each subject in the control group was calculated in TORTOISE using individual diffusion tensor images which had been aligned into the common tensor template space.

For datasets of the lesion group, the DWIs were also corrected for eddy current and EPI geometric distortion with the same method. Then, to perform voxel-wise comparisons between the control and lesion groups in the following TBSS analysis, the diffusion tensor images of each lesion subject were registered to the tensor template with the DRTAMAS tool in TORTOISE. After the diffusion tensor images of each subject in the lesion group were registered into the common tensor template space, the FA image of each subject in the lesion group was calculated. To minimize the potential misalignment caused by a lesion, a lesion-exclusion mask was implemented during registration.

### TBSS Analysis to Search for WM Voxels Showing Secondary Degeneration

Voxel-wise statistical analyses of the FA images between the control and lesion group were carried out using TBSS (Smith et al., 2006), part of FSL (Smith et al., 2004). The details of TBSS are provided in the notated reference (Smith et al., 2006) and are briefly discussed here. First, FA images of each subject in the control group were averaged to generate the mean FA images. Second, a skeletonized mean FA image was generated on the mean FA and thinned with a FA threshold of 0.3, the purpose of which was to represent the centers of all WM bundles. Third, each subject’s FA image was then projected onto the mean FA skeleton by searching the maximum value perpendicular to the local skeleton structure in the subject’s FA image. Following these steps, each subject’s FA images were aligned with the common skeleton. Voxel-wise differences between the control and lesion groups on the FA skeleton were compared using an unpaired *t*-test with threshold-free cluster enhancement (TFCE) (Smith and Nichols, 2009) and 3,003 permutation tests. Here, we considered *p* < 0.05 to be statistically significant after a permutation-based correction for multiple comparisons. Finally, detected skeleton voxels with statistical significance and their associated WM voxels were transformed back to each subject’s individual space for the next analysis of axonal connection properties of fibers passing through these voxels.

### Extraction of Sensorimotor Network Regions and Lesion Regions

After preprocessing, a rat brain MRI atlas (Sinke et al., 2018) matched with a three-dimensional model (Majka et al., 2012) of the Paxinos and Watson rat brain atlas (Paxinos and Watson, 2014) was non-linearly registered to the diffusion MRI data (*b* = 0 s/mm^2^) of each rat brain using a symmetric image normalization method (SyN) in ANTs (Avants et al., 2008). Then, brain regions in the sensorimotor network—including the primary motor cortex (M1), secondary motor cortex (M2), primary somatosensory cortex (S1), secondary somatosensory cortex (S2), caudate putamen (CPu), globus pallidus (GLO), and thalamus on both hemispheres—were extracted from the atlas. The lesion regions were manually drawn on *T*_2_-weighted images by one investigator on each subject in the lesion group. In addition, *T*_2_-weighted images of each lesioned subject were non-linearly registered to those of each control subject using SyN in ANTs (Avants et al., 2008) to identify the spatially matched ROI in the control group.

### Fiber Tracking

To further study which fibers enable secondary degeneration, whole-brain fiber tracking was performed in the individual space of each subject with deterministic streamline propagation using Euler methods (Yeh et al., 2013). Moreover, fibers through the TBSS-detected voxels were divided into two categories: fibers having direct connections with the primary lesion site (“direct” fibers) and fibers having no direct connections with the primary lesion (“indirect” fibers). Here, we defined “direct” fibers as fibers which start/terminate at the lesion site or passing through the lesion site and the “indirect” fibers as fibers which do not touch with the lesion site. Although we used the photothrombotic ischemic model to focus on induced lesion in the cortex, few WM lesions were observed. Considering that both cortical lesion and WM lesion could induce axonal secondary degeneration, we consequently defined both fibers starting/terminating at the lesion site (cortical lesion) and fibers passing through the lesion site (WM lesion) as “direct” fibers. All fiber tracking and the subsequent quantitative analysis of fibers were completed using DSI Studio software^2^. In detail, seeds were equally distributed in WM identified by a FA threshold of 0.3, and 10 million fibers were generated. The tracking was terminated when the FA was below 0.15 and the angle between two consecutive directions exceeded 55°. The TBSS-detected voxels with significantly decreased FA were transformed back to each subject’s individual space. Finally, fibers passing those voxels were selected in each subject for further analysis. The FA value of fibers considered to be microstructure metrics characterizing the fibers’ secondary degeneration was calculated by averaging FA values along the fibers. The sensorimotor network fibers were defined as fibers passing through the brain regions located in the sensorimotor network.

### Other Statistical Analyses

The median FA of fibers passing through the spatially matched ROI in six control subjects was used to perform an unpaired Student’s *t*-test with the FA of fibers in the lesion group; a *p* < 0.05 was considered to be statistically significant. The same method was used to compare the FA of fibers not passing through the lesion between the control and lesion groups. Finally, Spearman’s correlation tests were used to analyze the relationship between secondary WM degeneration (represented by the mean FA values of the TBSS-detected WM regions), primary lesion volume, and the motor behavioral score at 21 days after ischemia onset. These statistical analyses were completed in GraphPad Prism 8^3^.

## Results

### Structural Imaging Reveals Stable and Similar Primary Lesion Size and Locations in a PTI Rat Model

**FIGURE 2:**
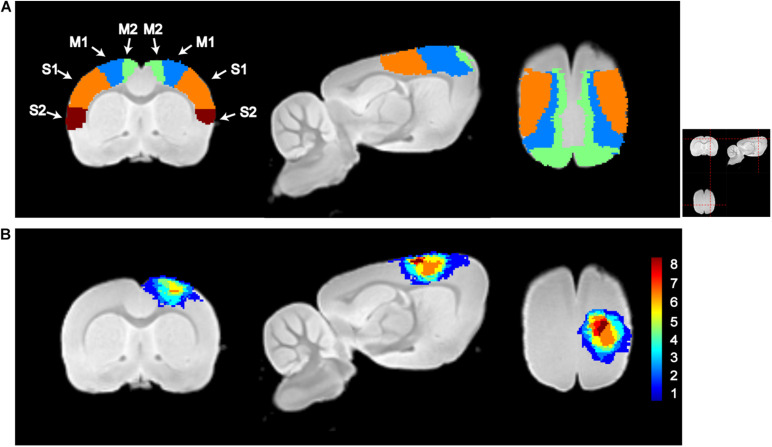
Illustration of sensorimotor cortex and lesion locations. **(A)** Segmentations of the primary motor cortex (M1), secondary motor cortex (M2), primary somatosensory cortex (S1), and secondary somatosensory motor cortex (S2). **(B)** Lesion maps of all subjects in the lesion group. The color in each voxel represents the number of rats with lesion in that voxel. M1, M2, S1, S2, and lesion maps were visualized by overlapping on the *T*_2_-weighted template, which was constructed using the structural images of the control group. From left to right, it is the coronal, sagittal, and axial views of the image. The red dashed lines in the top-right picture show the location for slices shown in **(A,B)**.

Figure 2 shows the sensorimotor cortex and overlying map of lesion on the template of *T*_2_-weighted images. As shown, the ischemic lesion size is relatively small with most voxels distributed in the sensorimotor cortex. The averaged lesion size is 19.2 ± 6.8 mm^3^ (mean ± *SD*). Lesion voxels are distributed in ipsilesional M1, M2, S1FL, and S1HL with the percentage of the injured volume in the total volume of this brain region 24.5 ± 12.1, 20.8 ± 11.9, 12.3 ± 17.3, and 18.9 ± 21.3%, respectively.

### TBSS Analysis Reveals Secondary Degeneration of Several WM Regions

**FIGURE 3:**
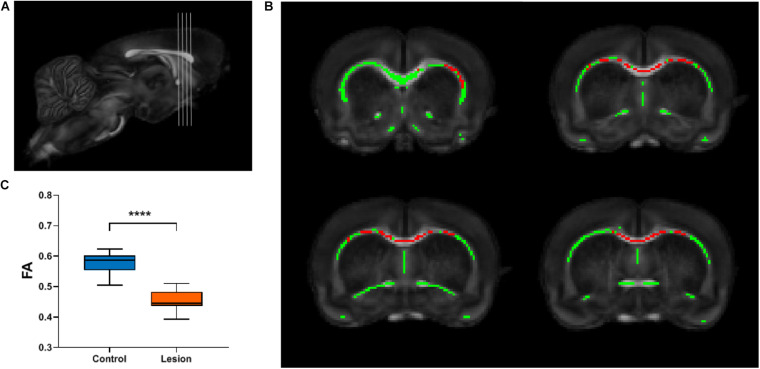
TBSS analysis reveals the white matter regions showing significant FA decreases in the lesion group. **(A)** Gray lines in the rat brain indicate different slices chosen for display in **(B)**. **(B)** The white matter skeleton is shown in green overlaid on the averaged FA map registered in a common space. Red color represents white matter skeleton with significant FA decrease in the lesion group compared with the control group (*p* < 0.05, multiple comparisons corrected). **(C)** Box plots of the FA values of the corresponding white matter regions in the individual space of the TBSS-detected skeleton regions shown in **(B)**. Student’s *t*-test was performed and **** means *p* < 0.0001.

To investigate whether focal cortical lesion could lead to secondary degeneration of WM distant to the lesions, we calculated differences in FA values of WM between the lesion group and the control group using TBSS for whole-brain analysis. Both the WM skeletons of the control and lesion groups are well constructed and aligned in the common space upon visual inspection, as shown in Figure 1C. Four slices shown in Figure 3A were chosen for displaying differences in FA between the lesion group and the control group. Figure 3B shows WM regions exhibiting significant decreases in FA in the lesion group (*p* < 0.05, TFCE corrected, labeled as red) primarily distributed in the ipsilesional external capsule and two remote regions including the anterior part of the corpus callosum (CC) and the contralesional external capsule. To further confirm secondary WM degeneration, these TBSS-detected ROIs were transformed back from the template space to the subject’s space using the transformation matrix obtained in the registration process. Not surprisingly, the mean FA values of those ROIs in the lesion group still indicated a significant decrease (*p* < 0.0001) compared with the control group (Figure 3C).

### Only Fibers Having Direct Connections With Primary Lesions Show Significant Microstructure Changes

**FIGURE 4:**
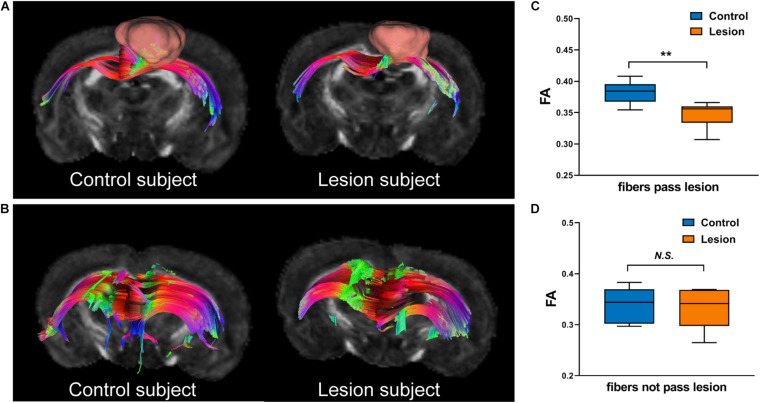
Illustration of fibers hosted by the TBSS-detected white matter regions. **(A)** “Direct” fibers that pass through the primary lesion (displayed with pink bulk) in the lesion group or the spatially matched ROI (displayed with pink bulk) in the control group. **(B)** “Indirect” fibers that do not pass through the primary lesion in the lesion group or the spatially matched ROI in the control group. **(C,D)** Boxplots of the FA values of direct fibers **(C)** and indirect fibers **(D)** in the control and lesion groups. Student’s *t*-test was performed here. **p < 0.01 and *^*N.S.*^p* > 0.05.

**FIGURE 5:**
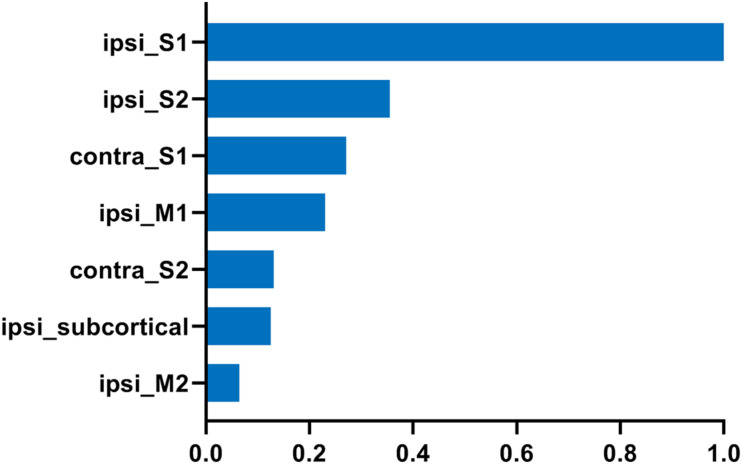
The top seven brain regions with the maximum number of direct fibers starting from or ending at in the control group. The direct fibers were the same as defined in Figure 4. The number of fibers was normalized to that of ipsilesional S1. “ipsi_” = ipsilesional and “contra_” = contralesional.

We further analyzed the axonal connection properties of fibers passing through the TBSS-detected WM voxels showing significant microstructure changes. We first studied those fibers passing the TBSS-detected WM voxels in the control group. Not surprisingly, we found that 90 ± 3% fibers belonged to the sensorimotor network. We further divided those sensorimotor network fibers into two categories: fibers having direct axonal connections with the primary lesion as shown in Figure 4A (“direct” fibers, i.e., fibers passing through the primary lesion) and fibers having no direct axonal connections with the primary lesion as shown in Figure 4B (“indirect” fibers). Interestingly, we found that only the direct fibers exhibited significant decreases in FA values (*p* = 0.0025, Figure 4C) when comparing the lesion group with the control group, whereas no significant differences were found on the indirect fibers (*p* = 0.696, Figure 4D). Figure 4A exhibits the fiber streamlines of the direct fibers, which mainly start from or end at the ipsilesional primary and secondary somatosensory cortex and some of the contralesional primary and secondary somatosensory cortex. Figure 5 provides the quantitative analysis of the axonal connection properties of these direct fibers in the control group and lists the top seven brain regions with the maximum number of direct fibers’ origin or termination. These brain regions correspond to the number of fibers: ipsilesional S1, ipsilesional S2, contralesional S1, ipsilesional M1, contralesional S2, ipsilesional subcortical areas, and ipsilesional M2. These findings suggest that the secondary WM degeneration primarily occurs on axonal connections with the ipsilesional somatosensory cortex and contralesional somatosensory cortex, with less occurrence on ipsilesional subcortical regions.

### Correlations Between Primary Lesion, Secondary Degeneration, and Motor Function Recovery

**FIGURE 6:**
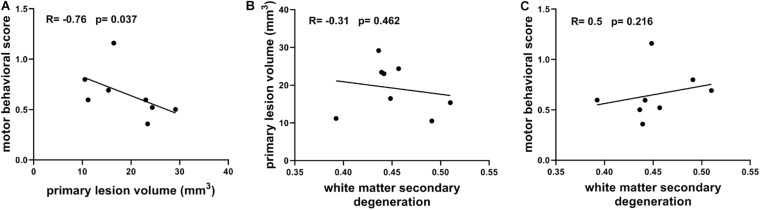
**(A)** Correlation between motor behavioral scores and primary lesion volume. **(B)** Correlation between primary lesion volume and white matter secondary degeneration which is characterized with the mean FA of TBSS-detected white matter regions. **(C)** Correlation between motor behavioral scores and white matter secondary degeneration. Spearman’s correlation tests were performed here with correlation coefficient (*R*) and *p*-values labeled.

Figure 6 shows the correlations between primary lesions, secondary degeneration, and motor function recovery, which were characterized using primary lesion volume, the mean FA values of the TBSS-detected WM regions (higher FA values correlate to lower degeneration), and the motor behavioral scores measured at 21 days after lesion induction, respectively. Interestingly, the primary lesion volumes show a significantly negative correlation with the motor behavioral scores (*R* = −0.76, *p* = 0.037, Figure 6A) and a positive but not significant correlation with the secondary degeneration of WM (Figure 6B). In contrast, the secondary degeneration of WM does not show a significant correlation (a trend in negative correlation) with the motor behavioral scores (*R* = 0.5, *p* = 0.216, Figure 6C).

## Discussion

In this study, we used high-resolution diffusion MRI and TBSS analysis methods to explore the secondary WM degeneration in a rat model of induced focal unilateral sensorimotor cortical ischemic lesions. A significant secondary degeneration was observed not only in the ipsilesional external capsule close to the primary lesions but also in remote regions including the CC and contralesional external capsule in the lesion group. More importantly, for fibers passing through these WM regions, only those having direct axonal connections with the primary lesion exhibited a significant secondary degeneration. In this study, we found the primary lesion volume might be the determining factor of the motor function recovery rather than the degree of secondary degeneration of WM.

TBSS provides a framework to search for voxel-wise changes in WM without any preliminary hypothesis by bringing different subjects from different groups into a common space for whole-brain comparison. Using the concept of the WM skeleton, in which the local maximum values in the individual FA map were projected to the group-mean FA skeleton, the false-positive findings in the edge of WM bundles induced by the misalignment in the registration process and the data smoothing steps could be reduced (Smith et al., 2006). In addition, we also chose to use the TFCE method, which is based on the permutation test, to further control the family-wise error rate for multiple comparison (Smith and Nichols, 2009). Furthermore, we improved TBSS analysis by replacing the conventional FA-based registration technique with diffeomorphic tensor-based registration with the SyN transformation model (Avants et al., 2008). Previous studies have shown that the use of full tensor features instead of tensor-derived indices, such as FA, for registration can significantly improve the alignment of WM tracts (Hecke et al., 2007; Hui et al., 2007) and further alleviate the impact of misassignments on TBSS results (Bach et al., 2014).

In this study, focal ischemic sensorimotor cortical lesions lead to secondary WM degeneration not only in the ipsilesional external capsule but also in regions distant to the primary lesion site, including the anterior part of the CC and the part of the external capsule in the contralesional hemisphere. The secondary degeneration of WM was characterized with decreases in FA values. The DTI-derived parameter FA measures water diffusion anisotropy (e.g., diffusion rate of water is faster along the direction parallel to fibers than the direction perpendicular to fibers) and is a highly sensitive index for detection of WM integrity properties (Assaf and Pasternak, 2008). Studies with histological validations demonstrate that decreased FA could reflect demyelination and axonal loss (Mac Donald et al., 2007; Kozlowski et al., 2008; Chandran et al., 2012; Cardenas et al., 2017). Previous studies suggest the decrease in FA in the chronic stage (after 3 weeks) mainly reflects chronic WM degeneration (Jung et al., 2017). Recently, studies with transmission electron microscopy also found the loss of structural integrity of axons in the WM bundles right below the sensorimotor cortex ischemic lesion site in a similar animal model (Rakib et al., 2019), which agrees well with the decreased FA in the ipsilesional external capsule that we found in this study.

Further investigation of the fibers passing through these TBSS-detected WM regions indicated, surprisingly, that secondary WM degeneration primarily occurs on fibers having direct axonal connections with the primary ischemic lesion (direct fibers) and does not expand to other fibers in the sensorimotor network having no direct axonal connections with the primary ischemic lesion (indirect fibers). Because TBSS only detects changes of FA in the WM skeleton whose values represent the maximum FA of all WM voxels perpendicular to the local skeleton, TBSS itself could not tell which fibers have significant degeneration and are the contributors of the skeleton changes. Further quantitative microstructure analysis along fibers demonstrated that the degeneration of direct fibers is the major contributor to the FA changes of TBSS-detected WM skeleton. The concept of secondary damage or degeneration of remote brain regions having direct structures tied to the primary lesion, named “diaschisis,” can be tracked back to the early twentieth century (Finger et al., 2004). Our results provide further imaging evidence supporting this concept by looking at the secondary degeneration of WM specifically in a whole-brain level. Recently, WM inflammation along axonal connectivity between sites of primary lesion and secondary degeneration was proposed as one of the key mechanisms of the secondary degeneration on remote cortical areas (Weishaupt et al., 2016). The same WM inflammation could explain the axonal degeneration on fibers having direct connections with the primary lesion, as inflammatory response and WM damage (such as demyelination or axonal loss) are closely related (Dos Santos et al., 2007; Souza-Rodrigues et al., 2008; Moxon-Emre and Schlichter, 2010).

In our results, focal unilateral ischemic lesions of the sensorimotor cortex induced secondary WM degeneration which primarily occurred on the perilesional axonal connections (ipsilesional M1, M2, S1, and S2) and the interhemispheric axonal connections (contralesional S1 and S2). Some also occurred on axonal connections with ipsilesional subcortical regions. The subregions in the sensorimotor cortex are strongly connected (van Meer et al., 2010; Gennaro et al., 2017), and it is not surprising that the axonal connections inside the sensorimotor cortex are damaged in this study. The finding of degeneration of fibers connecting the primary lesion site with the intact ipsilesional sensorimotor cortex agrees with a recent diffusion MRI study using graph theoretical analysis methods in a mouse stroke model (Pallast et al., 2020). The alteration of interhemispheric connectivity is a common phenomenon in most unilateral ischemic strokes, a finding which has been revealed using varying technologies such as resting-state functional MRI and manganese-enhanced MRI (van der Zijden et al., 2008; van Meer et al., 2010, 2012). Similar degeneration of interhemispheric fibers connecting lesioned areas with the sensorimotor cortex on the contralesional hemisphere has also recently been revealed by diffusion MRI in a photothrombotic stroke mouse model (Aswendt et al., 2020). Interhemispheric connectivity mediates the transfer of signals between hemispheres and plays important roles in sensorimotor function (van Meer et al., 2010, 2012). In addition, it has been revealed that fiber projections exist between the sensorimotor cortex and subcortical regions, such as the thalamus (Aldes, 1988; Kuramoto et al., 2009). The degeneration of WM connecting the primary lesion site with subcortical regions might explain the delayed neural cell loss in the thalamus after sensorimotor cortex ischemia, a finding which has been found in previous studies using similar animal models (Dihne et al., 2002; Schroeter et al., 2006).

In addition, we found that the primary lesion volume might be the major factor in determining the final motor function recovery level. This is not surprising. The correlation between final infarct volume and motor functional outcome at the chronic stage has been reported in other studies using a middle cerebral artery (MCAO) model in rats (Peeling et al., 2001; Roof et al., 2001; Turner et al., 2016). A similar dependence on the primary lesion volume of the final motor function outcome has also been found in human studies (Zaidi et al., 2012; Vagal et al., 2015). In some clinical studies, it has been reported that the secondary degeneration of WM shows good correlation with the functional outcome (Puig et al., 2010; Yin et al., 2013; Guo et al., 2017). However, in these clinical studies, the effect of infarct volume and localization on functional outcomes was not excluded in the statistical analysis considering the variety of lesion size and localization. In this study, when the primary lesion localization was well controlled and the variances in lesion volume were relatively small, the primary lesion was the major factor determining final motor deficits rather than secondary degeneration. Indeed, the correlation between secondary degeneration of WM and infarct volume was detected at the chronic stage after stroke in a previous study (Puig et al., 2010), and our results also show such a tendency. Considering the significant correlation between primary lesion and the motor function deficits, it is inferred that the primary lesion volume might be a factor of motor function deficits in this study. However, it also shows a trend in positive correlation between WM secondary degeneration and motor function deficit. On the whole, it could be that the primary lesion is the original determining factor of functional outcomes. Secondary degeneration could further contribute to functional outcomes but in a secondary role. Nevertheless, the interpretation for this finding should be more cautious in consideration of the relatively small sample size. In a future study, a larger sample size is needed to further explore the role of WM secondary degeneration and validate the role of primary lesion in motor function deficits.

Several limitations in this study and perspectives for future work should be considered. First, ideally, we should use sham animals by performing similar surgeries but with Rose Bengal solution replaced with saline solution as the control group (Okabe et al., 2016). The purpose for such sham control design is to rule out the potential side effects of the surgery itself, which mainly refers to the potential effects of laser illumination on animals and the potential artifacts on MRI due to skull removal procedure. However, a previous study used the same photothrombotic ischemic model in rats and showed no abnormal intensity in MRI images, no changes in cerebral blood flow, and no behavioral deficit in the sham group with saline injection and illumination compared with healthy controls without any surgery (Lu et al., 2014). In addition, we performed *ex vivo* MRIs on rat brains without skulls, which could further avoid potential MRI artifacts. Second, the dynamic observation on WM microstructure changes is lacking in this study. Besides, the sample size is relatively small. In the future, a longitudinal study combined with a larger sample size will help us further explore the evolution of secondary degeneration of WM and its dynamic relation with motor functions. What is more, functional information of connectivity would help to explore the mechanisms underlying microstructure changes and motor function outcome. In the future, longitudinal studies combined with diffusion MRI and functional MRI are planned. Due to the inherent technological limitation of diffusion tensor imaging, it cannot make a distinction between two parallel but opposite directions. In this study, WM secondary degeneration was found after focal ischemic lesion. However, the anterograde and retrograde degeneration could not be differentiated by diffusion tensor image in this study when the projection between the lesion site and remote intact sensorimotor cortex is bidirectional. It will be an interesting direction to research anterograde and retrograde degeneration along a unidirectional projection pathway in a future study. Finally, although the histological results from similar animal models support our findings, it would be desirable to compare MRI findings with histological findings in the future.

## Conclusion

In conclusion, by using high-resolution MRI, advanced whole-brain analysis methods, and well-controlled ischemic lesion animal models, we found broad secondary degeneration in white matter following focal ischemic lesions in the sensorimotor cortex. More importantly, secondary degeneration primarily occurs on fibers having direct axonal connections with the primary lesion, which includes the perilesional, interhemispheric, and ipsilesional subcortical axonal connections. The primary lesion volume plays the most important role in determining final motor function deficits.

## Data Availability Statement

The raw data supporting the conclusions of this article will be made available by the authors, without undue reservation.

## Ethics Statement

The animal study was reviewed and approved by the Guide for The Care and Use of Laboratory Animals (China Ministry of Health) and the Animal Care Committee of Zhejiang University, China.

## Author Contributions

ZL, HG, KX, and RB designed the study and contributed to the data interpretation and the manuscript. HG and YJ performed the animal experiments. PZ and XK conducted the MRI scanning. ZL performed the data analysis. All authors contributed to the article and approved the submitted version.

## Conflict of Interest

The authors declare that the research was conducted in the absence of any commercial or financial relationships that could be construed as a potential conflict of interest.

## Footnotes

1 https://mipav.cit.nih.gov/

2 http://dsi-studio.labsolver.org

3 https://www.graphpad.com/scientific-software/prism/

## References

[B1] Alaverdashvili M. Whishaw I. Q. (2013). A behavioral method for identifying recovery and compensation: hand use in a preclinical stroke model using the single pellet reaching task. *Neurosci. Biobehav. Rev.* 37 950–967. 10.1016/j.neubiorev.2013.03.026 23583614

[B2] Aldes L. D. (1988). Thalamic connectivity of rat somatic motor cortex. *Brain Res. Bull.* 20 333–348. 10.1016/0361-9230(88)90063-9 2452673

[B3] Alexander D. C. Dyrby T. B. Nilsson M. Zhang H. (2019). Imaging brain microstructure with diffusion MRI: practicality and applications. *NMR Biomed.* 32:e3841. 10.1002/nbm.3841 29193413

[B4] Assaf Y. Pasternak O. (2008). Diffusion tensor imaging (DTI)-based white matter mapping in brain research: a review. *J. Mol. Neurosci.* 34 51–61. 10.1007/s12031-007-0029-0 18157658

[B5] Aswendt M. Pallast N. Wieters F. Baues M. Hoehn M. Fink G. R. (2020). *Lesion Size- and Location-Dependent Recruitment of Contralesional Thalamus and Motor Cortex Facilitates Recovery after Stroke in Mice. Translational Stroke Research.* New York: Springer. 10.1007/s12975-020-00802-3 PMC7803721 32166716

[B6] Avants B. B. Epstein C. L. Grossman M. Gee J. C. (2008). Symmetric diffeomorphic image registration with cross-correlation: evaluating automated labeling of elderly and neurodegenerative brain. *Med. Image Anal.* 12 26–41. 10.1016/j.media.2007.06.004 17659998 PMC2276735

[B7] Bach M. Laun F. B. Leemans A. Tax C. M. Biessels G. J. Stieltjes B. (2014). Methodological considerations on tract-based spatial statistics (TBSS). *Neuroimage* 100 358–369. 10.1016/j.neuroimage.2014.06.021 24945661

[B8] Basser P. J. Mattiello J. LeBihan D. (1994). MR diffusion tensor spectroscopy and imaging. *Biophys. J.* 66 259–267. 10.1016/S0006-3495(94)80775-1 8130344 PMC1275686

[B9] Cao Z. Harvey S. S. Bliss T. M. Cheng M. Y. Steinberg G. K. (2020). Inflammatory Responses in the Secondary Thalamic Injury After Cortical Ischemic Stroke. *Front. Neurol.* 11:236 10.3389/fneur.2020.00236 PMC7154072 32318016

[B10] Cardenas A. M. Sarlls J. E. Kwan J. Y. Bageac D. Gala Z. S. Danielian L. E. (2017). Pathology of callosal damage in ALS: An ex-vivo, 7T diffusion tensor MRI study. *NeuroImage Clin.* 15 200–208. 10.1016/j.nicl.2017.04.024 28529876 PMC5429246

[B11] Chandran P. Upadhyay J. Markosyan S. Lisowski A. Buck W. Chin C. L. (2012). Magnetic resonance imaging and histological evidence for the blockade of cuprizone-induced demyelination in C57BL/6 mice. *Neuroscience* 202 446–453. 10.1016/j.neuroscience.2011.10.051 22119061

[B12] Dancause N. Barbay S. Frost S. B. Zoubina E. V. Plautz E. J. Mahnken J. D. (2006). Effects of small ischemic lesions in the primary motor cortex on neurophysiological organization in ventral premotor cortex. *J. Neurophysiol.* 96 3506–3511. 10.1152/jn.00792.2006 16987930

[B13] Dihne M. Grommes C. Lutzenburg M. Witte O. W. Block F. (2002). Different mechanisms of secondary neuronal damage in thalamic nuclei after focal cerebral ischemia in rats. *Stroke* 33 3006–3011. 10.1161/01.str.0000039406.64644.cb 12468804

[B14] Dikranian K. Cohen R. Mac Donald C. Pan Y. Brakefield D. Bayly P. (2008). Mild traumatic brain injury to the infant mouse causes robust white matter axonal degeneration which precedes apoptotic death of cortical and thalamic neurons. *Exp. Neurol.* 211 551–560. 10.1016/j.expneurol.2008.03.012 18440507 PMC2486437

[B15] Dos Santos C. D. Picanço-Diniz C. W. Gomes-Leal W. (2007). Differential patterns of inflammatory response, axonal damage and myelin impairment following excitotoxic or ischemic damage to the trigeminal spinal nucleus of adult rats. *Brain Res.* 1172 130–144. 10.1016/j.brainres.2007.07.037 17822682

[B16] Duering M. Righart R. Wollenweber F. A. Zietemann V. Gesierich B. Dichgans M. (2015). Acute infarcts cause focal thinning in remote cortex via degeneration of connecting fiber tracts. *Neurology* 84:1685. 10.1212/WNL.0000000000001502 25809303 PMC4409580

[B17] Egorova N. Dhollander T. Khlif M. S. Khan W. Werden E. Brodtmann A. (2020). Pervasive White Matter Fiber Degeneration in Ischemic Stroke. *Stroke* 51 1507–1513. 10.1161/STROKEAHA.119.028143 32295506

[B18] Finger S. Koehler P. J. Jagella C. (2004). The Monakow concept of diaschisis: origins and perspectives. *Arch. Neurol.* 61 283–288. 10.1001/archneur.61.2.283 14967781

[B19] Gennaro M. Mattiello A. Mazziotti R. Antonelli C. Gherardini L. Guzzetta A. (2017). Focal Stroke in the Developing Rat Motor Cortex Induces Age- and Experience-Dependent Maladaptive Plasticity of Corticospinal System. *Front. Neural. Circ.* 11:47. 10.3389/fncir.2017.00047 28706475 PMC5489564

[B20] Guo A. H. Hao F. L. Liu L. F. Wang B. J. Jiang X. F. (2017). An assessment of the correlation between early postinfarction pyramidal tract Wallerian degeneration and nerve function recovery using diffusion tensor imaging. *Genet Mol. Res.* 16:23. 10.4238/gmr16019035 28128402

[B21] Haber M. Hutchinson E. B. Sadeghi N. Cheng W. H. Namjoshi D. Cripton P. (2017). Defining an Analytic Framework to Evaluate Quantitative MRI Markers of Traumatic Axonal Injury: Preliminary Results in a Mouse Closed Head Injury Model. *eNeuro* 4 ENEURO.164–117. 10.1523/ENEURO.0164-17.2017 28966972 PMC5616192

[B22] Hecke W. V. Leemans A. Agostino E. D. Backer S. D. Vandervliet E. Parizel P. M. (2007). Nonrigid Coregistration of Diffusion Tensor Images Using a Viscous Fluid Model and Mutual Information. *IEEE Trans. Med. Imaging* 26 1598–1612. 10.1109/TMI.2007.906786 18041274

[B23] Hui Z. Avants B. B. Yushkevich P. A. Woo J. H. Sumei W. McCluskey L. F. (2007). High-Dimensional Spatial Normalization of Diffusion Tensor Images Improves the Detection of White Matter Differences: An Example Study Using Amyotrophic Lateral Sclerosis. *IEEE Trans. Med. Imaging* 26 1585–1597. 10.1109/tmi.2007.906784 18041273

[B24] Irfanoglu M. O. Modi P. Nayak A. Hutchinson E. B. Sarlls J. Pierpaoli C. (2015). DR-BUDDI (Diffeomorphic Registration for Blip-Up blip-Down Diffusion Imaging) method for correcting echo planar imaging distortions. *Neuroimage* 106 284–299. 10.1016/j.neuroimage.2014.11.042 25433212 PMC4286283

[B25] Irfanoglu M. O. Nayak A. Jenkins J. Hutchinson E. B. Sadeghi N. Thomas C. P. (2016). DR-TAMAS: Diffeomorphic Registration for Tensor Accurate Alignment of Anatomical Structures. *Neuroimage* 132 439–454. 10.1016/j.neuroimage.2016.02.066 26931817 PMC4851878

[B26] Jian Z. Liu R. Zhu X. Smerin D. Zhong Y. Gu L. (2019). The Involvement and Therapy Target of Immune Cells After Ischemic Stroke. *Front. Immunol.* 10 2167–2167. 10.3389/fimmu.2019.02167 31572378 PMC6749156

[B27] Jiang Q. Zhang Z. G. Ding G. L. Silver B. Zhang L. Meng H. (2006). MRI detects white matter reorganization after neural progenitor cell treatment of stroke. *NeuroImage* 32 1080–1089. 10.1016/j.neuroimage.2006.05.025 16860575

[B28] Jones K. A. Zouikr I. Patience M. Clarkson A. N. Isgaard J. Johnson S. J. (2015). Chronic stress exacerbates neuronal loss associated with secondary neurodegeneration and suppresses microglial-like cells following focal motor cortex ischemia in the mouse. *Brain Behav. Immun.* 48 57–67. 10.1016/j.bbi.2015.02.014 25749481

[B29] Jung W. B. Han Y. H. Chung J. J. Chae S. Y. Lee S. H. Im G. H. (2017). Spatiotemporal microstructural white matter changes in diffusion tensor imaging after transient focal ischemic stroke in rats. *NMR Biomed.* 30:e3704. 10.1002/nbm.3704 28205341

[B30] Kozlowski P. Raj D. Liu J. Lam C. Yung A. C. Tetzlaff W. (2008). Characterizing white matter damage in rat spinal cord with quantitative MRI and histology. *J. Neurotrauma.* 25 653–676. 10.1089/neu.2007.0462 18578635

[B31] Krishnamurthi R. V. Moran A. E. Feigin V. L. Barker-Collo S. Norrving B. Mensah G. A. (2015). Stroke Prevalence, Mortality and Disability-Adjusted Life Years in Adults Aged 20-64 Years in 1990-2013: Data from the Global Burden of Disease 2013 Study. *Neuroepidemiology* 45 190–202. 10.1159/000441098 26505983

[B32] Kuramoto E. Furuta T. Nakamura K. C. Unzai T. Hioki H. Kaneko T. (2009). Two Types of Thalamocortical Projections from the Motor Thalamic Nuclei of the Rat: A Single Neuron-Tracing Study Using Viral Vectors. *Cerebral Cortex* 19 2065–2077. 10.1093/cercor/bhn231 %J Cerebral Cortex 19174446

[B33] Labat-gest V. Tomasi S. (2013). Photothrombotic ischemia: a minimally invasive and reproducible photochemical cortical lesion model for mouse stroke studies. *J. Vis. Exp.* 76:50370. 10.3791/50370 23770844 PMC3727176

[B34] Laule C. Vavasour I. M. Kolind S. H. Li D. K. B. Traboulsee T. L. Moore G. R. W. (2007). Magnetic resonance imaging of myelin. *Neurotherapeutics* 4 460–484. 10.1016/j.nurt.2007.05.004 17599712 PMC7479725

[B35] Lindberg P. G. Skejø P. H. B. Rounis E. Nagy Z. Schmitz C. Wernegren H. (2007). Wallerian Degeneration of the Corticofugal Tracts in Chronic Stroke: A Pilot Study Relating Diffusion Tensor Imaging, Transcranial Magnetic Stimulation, and Hand Function. *Neurorehabil. Neural. Repair* 21 551–560. 10.1177/1545968307301886 17507645

[B36] Lu H. Li Y. Yuan L. Li H. Lu X. Tong S. (2014). Induction and imaging of photothrombotic stroke in conscious and freely moving rats. *J. Biomed. Opt.* 19:96013 10.1117/1.JBO.19.9.096013 25239673

[B37] Mac Donald C. L. Dikranian K. Song S. K. Bayly P. V. Holtzman D. M. Brody D. L. (2007). Detection of traumatic axonal injury with diffusion tensor imaging in a mouse model of traumatic brain injury. *Exp. Neurol.* 205 116–131. 10.1016/j.expneurol.2007.01.035 17368446 PMC1995439

[B38] Majid A. (2014). Neuroprotection in stroke: past, present, and future. *ISRN Neurol.* 2014 515716–515716. 10.1155/2014/515716 24579051 PMC3918861

[B39] Majka P. Kublik E. Furga G. Wojcik D. K. (2012). Common atlas format and 3D brain atlas reconstructor: infrastructure for constructing 3D brain atlases. *Neuroinformatics* 10 181–197. 10.1007/s12021-011-9138-6 22227717 PMC3325030

[B40] Moxon-Emre I. Schlichter L. C. (2010). Evolution of inflammation and white matter injury in a model of transient focal ischemia. *J. Neuropathol. Exp. Neurol.* 69 1–15. 10.1097/NEN.0b013e3181c3ce6c 20010307

[B41] Mukherjee P. Chung S. W. Berman J. I. Hess C. P. Henry R. G. (2008). Diffusion tensor MR imaging and fiber tractography: technical considerations. *AJNR Am. J. Neuroradiol.* 29 843–852. 10.3174/ajnr.A1052 18339719 PMC8128579

[B42] Neher P. Stieltjes B. Wolf I. Meinzer H. Maier-Hein K. (2013). “Analysis of tractography biases introduced by anisotropic voxels,” in *Proc. Annual Meeting ISMRM*, (Germany: ISMRM).

[B43] Nudo R. J. (2006). Mechanisms for recovery of motor function following cortical damage. *Curr. Opin. Neurobiol.* 16 638–644. 10.1016/j.conb.2006.10.004 17084614

[B44] Okabe N. Shiromoto T. Himi N. Lu F. Maruyama-Nakamura E. Narita K. (2016). Neural network remodeling underlying motor map reorganization induced by rehabilitative training after ischemic stroke. *Neuroscience* 339 338–362. 10.1016/j.neuroscience.2016.10.008 27725217

[B45] Pallast N. Wieters F. Nill M. Fink G. R. Aswendt M. (2020). Graph theoretical quantification of white matter reorganization after cortical stroke in mice. *Neuroimage* 217:116873. 10.1016/j.neuroimage.2020.116873 32380139

[B46] Pantoni L. Garcia J. H. Gutierrez J. A. (1996). Cerebral white matter is highly vulnerable to ischemia. *Stroke* 27 1641–1646. 10.1161/01.str.27.9.1641 8784142

[B47] Paxinos G. Watson C. (2014). *The rat brain in stereotaxic coordinates.* Amsterdam: Elsevier Academic Press.

[B48] Peeling J. Corbett D. Del Bigio M. R. Hudzik T. J. Campbell T. M. Palmer G. C. (2001). Rat middle cerebral artery occlusion: correlations between histopathology, T2-weighted magnetic resonance imaging, and behavioral indices. *J. Stroke Cerebrovasc. Dis.* 10 166–177. 10.1053/jscd.2001.26865 17903821

[B49] Pierpaoli C. Walker L. Irfanoglu M. Barnett A. Basser P. Chang L.-C. (2010). “TORTOISE: an integrated software package for processing of diffusion MRI data,” in *ISMRM 18th Annual Meeting*, (Germany: ISMRM).

[B50] Puig J. Pedraza S. Blasco G. Daunis I. E. J. Prats A. Prados F. (2010). Wallerian degeneration in the corticospinal tract evaluated by diffusion tensor imaging correlates with motor deficit 30 days after middle cerebral artery ischemic stroke. *AJNR Am. J. Neuroradiol.* 31 1324–1330. 10.3174/ajnr.A2038 20299434 PMC7965455

[B51] Rakib F. Ali C. M. Yousuf M. Afifi M. Bhatt P. R. Ullah E. (2019). Investigation of Biochemical Alterations in Ischemic Stroke Using Fourier Transform Infrared Imaging Spectroscopy-A Preliminary Study. *Brain Sci.* 9:293. 10.3390/brainsci9110293 31717715 PMC6895834

[B52] Roof R. L. Schielke G. P. Ren X. Hall E. D. (2001). A comparison of long-term functional outcome after 2 middle cerebral artery occlusion models in rats. *Stroke* 32 2648–2657. 10.1161/hs1101.097397 11692030

[B53] Schroeter M. Zickler P. Denhardt D. T. Hartung H.-P. Jander S. (2006). Increased thalamic neurodegeneration following ischaemic cortical stroke in osteopontin-deficient mice. *Brain* 129 1426–1437. 10.1093/brain/awl094 16636021

[B54] Sinke M. R. T. Otte W. M. Christiaens D. Schmitt O. Leemans A. van der Toorn A. (2018). Diffusion MRI-based cortical connectome reconstruction: dependency on tractography procedures and neuroanatomical characteristics. *Brain Struct. Funct.* 223 2269–2285. 10.1007/s00429-018-1628-y 29464318 PMC5968063

[B55] Smith S. M. Nichols T. E. (2009). Threshold-free cluster enhancement: addressing problems of smoothing, threshold dependence and localisation in cluster inference. *Neuroimage* 44 83–98. 10.1016/j.neuroimage.2008.03.061 18501637

[B56] Smith S. M. Jenkinson M. Johansen-Berg H. Rueckert D. Nichols T. E. Mackay C. E. (2006). Tract-based spatial statistics: voxelwise analysis of multi-subject diffusion data. *Neuroimage* 31 1487–1505. 10.1016/j.neuroimage.2006.02.024 16624579

[B57] Smith S. M. Jenkinson M. Woolrich M. W. Beckmann C. F. Behrens T. E. J. Johansen-Berg H. (2004). Advances in functional and structural MR image analysis and implementation as FSL. *NeuroImage* 23 S208–S219. 10.1016/j.neuroimage.2004.07.051 15501092

[B58] Souza-Rodrigues R. D. Costa A. M. R. Lima R. R. Dos Santos C. D. Picanço-Diniz C. W. Gomes-Leal W. (2008). Inflammatory response and white matter damage after microinjections of endothelin-1 into the rat striatum. *Brain Res.* 1200 78–88. 10.1016/j.brainres.2007.11.025 18289508

[B59] Stroemer R. P. Kent T. A. Hulsebosch C. E. (1995). Neocortical neural sprouting, synaptogenesis, and behavioral recovery after neocortical infarction in rats. *Stroke* 26 2135–2144. 10.1161/01.str.26.11.2135 7482662

[B60] Turner R. C. DiPasquale K. Logsdon A. F. Tan Z. Naser Z. J. Huber J. D. (2016). The role for infarct volume as a surrogate measure of functional outcome following ischemic stroke. *J. Syst. Integr. Neurosci.* 2:10.15761/JSIN.1000136. 10.15761/JSIN.1000136 28299202 PMC5347398

[B61] Vagal A. S. Sucharew H. Prabhakaran S. Khatri P. Jovin T. Michel P. (2015). Final infarct volume discriminates outcome in mild strokes. *Neuroradiol. J.* 28 404–408. 10.1177/1971400915609347 26427891 PMC4757309

[B62] van der Zijden J. P. Bouts M. J. Wu O. Roeling T. A. Bleys R. L. van der Toorn A. (2008). Manganese-enhanced MRI of brain plasticity in relation to functional recovery after experimental stroke. *J. Cereb. Blood Flow Metab.* 28 832–840. 10.1038/sj.jcbfm.9600576 17987047

[B63] van Meer M. P. Otte W. M. van der Marel K. Nijboer C. H. Kavelaars A. van der Sprenkel J. W. (2012). Extent of bilateral neuronal network reorganization and functional recovery in relation to stroke severity. *J. Neurosci.* 32 4495–4507. 10.1523/JNEUROSCI.3662-11.2012 22457497 PMC6622065

[B64] van Meer M. P. van der Marel K. Wang K. Otte W. M. El Bouazati S. Roeling T. A. (2010). Recovery of sensorimotor function after experimental stroke correlates with restoration of resting-state interhemispheric functional connectivity. *J. Neurosci.* 30 3964–3972. 10.1523/JNEUROSCI.5709-09.2010 20237267 PMC6632290

[B65] Wei X. E. Shang K. Zhou J. Zhou Y. J. Li Y. H. (2019). Acute Subcortical Infarcts Cause Secondary Degeneration in the Remote Non-involved Cortex and Connecting Fiber Tracts. *Front. Neurol.* 10:860. 10.3389/fneur.2019.00860 31440202 PMC6693082

[B66] Weishaupt N. Zhang A. Deziel R. A. Tasker R. A. Whitehead S. N. (2016). Prefrontal Ischemia in the Rat Leads to Secondary Damage and Inflammation in Remote Gray and White Matter Regions. *Front. Neurosci.* 10:81. 10.3389/fnins.2016.00081 26973455 PMC4773446

[B67] Yeh F.-C. Verstynen T. D. Wang Y. Fernández-Miranda J. C. Tseng W.-Y. I. (2013). Deterministic Diffusion Fiber Tracking Improved by Quantitative Anisotropy. *PLoS One* 8:e80713. 10.1371/journal.pone.0080713 24348913 PMC3858183

[B68] Yin D. Yan X. Fan M. Hu Y. Men W. Sun L. (2013). Secondary degeneration detected by combining voxel-based morphometry and tract-based spatial statistics in subcortical strokes with different outcomes in hand function. *AJNR Am. J. Neuroradiol.* 34 1341–1347. 10.3174/ajnr.A3410 23391838 PMC8051511

[B69] Yu C. Zhu C. Zhang Y. Chen H. Qin W. Wang M. (2009). A longitudinal diffusion tensor imaging study on Wallerian degeneration of corticospinal tract after motor pathway stroke. *NeuroImage* 47 451–458. 10.1016/j.neuroimage.2009.04.066 19409500

[B70] Zaidi S. F. Aghaebrahim A. Urra X. Jumaa M. A. Jankowitz B. Hammer M. (2012). Final infarct volume is a stronger predictor of outcome than recanalization in patients with proximal middle cerebral artery occlusion treated with endovascular therapy. *Stroke* 43 3238–3244. 10.1161/STROKEAHA.112.671594 23160876

